# Evaluation of IL-12A, IL-12B, IL-23A and IL-27 mRNA expression level genes in peripheral mononuclear cells of inflammatory bowel disease patients in an Iranian population 

**Published:** 2018

**Authors:** Mohsen Norouzinia, Vahid Chaleshi, Samaneh Alinaghi, Saeedeh sadat Beheshti Shirazi, Aliasghar Keramatinia, Mahyar Nourian

**Affiliations:** 1 *Gastroenterology and Liver Diseases Research Center, Research Institute for Gastroenterology and Liver Diseases, Shahid Beheshti University of Medical Sciences, Tehran, Iran *; 2 *Basic and Molecular Epidemiology of Gastrointestinal Disorders Research Center, Research Institute for Gastroenterology and Liver Diseases, Shahid Beheshti University of Medical Sciences, Tehran, Iran *; 3 *Foodborne and Waterborne Diseases Research Center, Research Institute for Gastroenterology and Liver Diseases, Shahid Beheshti University of Medical Sciences, Tehran, Iran *; 4 *Proteomics Research Center, Faculty of Paramedical Sciences, Shahid Beheshti University of Medical Sciences, Tehran, Iran*

**Keywords:** Flare-up phase, Inflammatory bowel disease, Interleukins, Remission phase, Interleukins, Gene expression

## Abstract

**Aim:**

Aim of this study was to compare the gene expression of Interleukin 12 members in two phase of IBD.

**Background:**

Inflammatory bowel disease (IBD) is a well-known gastrointestinal disorder in the world that fluctuates between remission and flare-up phases. Each of these phases has an individual immune system response profile. Therefore, analyzing the interleukins (IL) expression status improves the diagnosis and the classification of the IBD cases.

**Methods:**

In this a case-control study, among 400 patients whom admitted to the IBD clinic, forty nine IBD patients were included. Patients were divided into three categories based on 1) the phase of the disease, 2) the type of IBD, Ulcerative colitis (UC) or Crohn's disease (CD), and 3) the therapeutic pathways. Using the real-time PCR method, the expression levels of IL-12A, IL-12B, IL-23A, and IL-27 were examined in the peripheral blood mononuclear cell (PBMC) and compared to the pre-described subgroups.

**Results:**

the data showed upregulation in the expression levels of IL-12A and IL-12B in the remission phase in comparison with the flare-up. However, no significant changes were obtained from the evaluation of IL-23A and IL-27. In addition, the mRNA levels of the target genes in the subgroups of Category 2 as well as Category 3 were similar.

**Conclusion:**

Our results showed that expression patterns of the IL-12A and IL-12B genes varied between the remission and flare-up phases for the IBD patients, and may be considered as potential biomarkers for the detection and the classification of IBD cases.

## Introduction

 Inflammatory bowel disease (IBD) is caused via dysregulation of the immune system inducing the production of proinflammatory cytokines and adhesion molecules (1). The main cause of IBD remains unclear. Crohn's disease (CD) and ulcerative colitis (UC) are the two deferent types of IBD. Previous studies have clarified that genetic and environments' predisposition to IBD may exist. IBD may be caused by immune system oversight in response to microflora and/or some of the environmental factors that affect genetically susceptible individuals. Genetic studies also have demonstrated the role of host-microflora associations in the IBD progression (2). Idiopathic IBD comprises of UC and CD, the two major chronic intestinal disorders (1-3). UC is marked by the chronic inflammation of the colon and rectum, whereas in CD cases the whole gastrointestinal tract is involved (3). These complications could disrupt the quality of lifestyle of the patients and impose enormous social and economic burden on the society (1). As major players in the inflammation process, cytokines mediate the immune responses. Interleukins (ILs) are the known clusters of cytokines, which regulate the proliferation, development, and activation of the immune cells. However, the different aspects of a particular interleukin function are dependent on the type of cytokine producing cell and/or the target organ. Due to this reason has not been completely elucidated yet. During recent years, the actions of multiple cytokines, such as IL-1, IL-5, IL-6, IL-10, IL-12, IL-13, IL-16, IL-17, IL-18, IL-21, IL-22, IL-23, IL-27, IL-33, IL-35, and IL-37, have been reported in the progression of IBD complications. However, many studies for underlying mechanisms are ongoing (2, 4, 5). The interleukin 12 (IL-12) cytokine family includes IL-12, IL-23, IL-27 (IL12A OMIM: 161560, IL12B OMIM: 161561, IL23A OMIM: 605580, IL27 OMIM: 608273) and its most recent member, IL-35. Each cytokine is formed of two subunits, some of which form bioactive cytokines themselves, and others which are shared among family members. These cytokines with a great degree of interaction potential not present in other cytokine families, both in the context of receptor binding and other molecular interactions. This diversity is perhaps responsible for the highly variable biological functions that have been reported in the context of preclinical studies of inflammation. Given the overlapping structural components and diversification of function, targeting the IL-12 cytokine family in the context of health and disease requires in depth knowledge and highly specific treatments (6). IL- 12 is predominantly a pro-inflammatory cytokine secreted APC in response to sensing of microbial components by Toll like receptors (7). The most notorious function of IL-12 is the induction of T-bet and control of the differentiation of naive T cells into IFN-g-producing Th1 cells. IL-12 has a potent inducer of IFNg by natural killer cells and innate lymphoid cells (ILCs). Another member of this family, IL-23 is also produced by DCs and macrophages after TLR engagement. Expression of IL-23 is further augmented by interactions after CD40 activation. Regulation of the IL-23R is controlled by the cytokines IL-6 and IL-21 (8). A positive feedback loop is then created, in which IL-23 expression from APCs drives the expansion of these licensed, proliferating T cells (9). IL-27 signals through a receptor composed of gp130, the common IL-6 receptor chain, and a unique IL-27 receptor a-chain (IL-27RA) that shares homology with the IL-12Rb2 chain of the IL- 12 receptor. IL-27 was initially thought to induce inflammation, however new evidence shows that IL-27 possesses immunoregulatory capabilities (10).

In patients suffering from UC, as well as the CD patients, the levels of inflammatory cytokines are different from that of the healthy individuals. Previous epidemiological studies on IBD patients have showed an increment in IL-16, at the mRNA and protein level, in the pathologic region of the intestinal and blood serum (2, 11-13). Thereby, as demonstrated in the experimental rats, blocking the IL-16 would improve the colitis-like inflammation (2, 14, 15). Also, growing evidence suggest a linkage between the polymorphism rate of the promoter region of interleukins genes and CD progression (2, 16). 

One of the major areas of attention in the case of IBD patients is the shifting of the current status of their disease from remission into the flare-up phase (17). Therefore, the aim of this study is to evaluate the expression pattern of four interleukins genes; IL-12A, IL-12B, IL-23A, and IL-27 in IBD patients and compared their levels in the remission and active phases. Also, the mRNA levels of these genes were compared separately, based on whether the patients had UC or CD, and according to the type of drugs they were treated with. 

## Methods

**Study population**

The patients included this study from 400 patients whom admitted to the IBD clinic of the Research institute for Gastroenterology and Liver Diseases (RIGLD) of Shahid Beheshti University of Medical Sciences Tehran, Iran (SBMU) between December 2017 and June 2018. All the participants were well-informed about the tests progress and their clinical data was obtained by reviewing and completing a standard questionnaire application. All of IBD patients for whom have done colonoscopy the tissue specimens were sampled. Expert pathologist confirmed the UC or CD. The severity of disease and the current phase of the disease was evaluated by all of clinical, radiologic and pathologic data base on Ulcerative colitis activity index and Crohn's disease activity index. Finally 49 IBD patients were included in the study. The demographic data of the patients, such as age, sex, weight, body mass index (BMI), and familial history of IBD, and their drug intake status were taken from the clinic records. The methods were approved by the RIGLD's ethics committee. In this study The inclusions criteria were; 1-IBD patients who were originally from Iran, 2-Cases with positive IBD pathology results, and also the exclusion criteria were; 1-Individuals that underwent colonoscopy, but the biopsy was not performed completely. 2-Those who did not have valid clinical and pathological data such as indeterminate colitis, post-radiation colitis, infectious colitis and others, or patients with no pathological samples through colonoscopy.

The patients were divided into the following subgroups and compared based on the phase of the disease; the remission or flare-up, whether they had UC or CD, and finally according to the therapeutic medicines: 5-aminosalicylic acid (5ASA), prednisone (Pred), Azathioprine (AZA), and Infliximab (IFX).

**Blood Sampling**

Up to 4 ml of whole blood was collected from each of the IBD patients and stored in EDTA tubes. For peripheral blood mononuclear cells (PBMC) separation, the samples were at first diluted with PBS in the ratio of 1:1 (v/v). Then, 3 ml of Ficoll buffer was gently added to the mixture and the tube was centrifuged at 1000 g for 15 min to separate the blood's phases. The obtained PBMC samples were washed with PBS and subsequently stored in aliquots at −20°C, until use.

**RNA extraction and real-time PCR analysis**

Total RNA was extracted from the PBMC of the patients using the Total RNA Extraction Mini Kit (Yekta Tajhiz Co, Tehran,Iran), as per the manufacturer’s instructions. The RNA concentration was measured by a Nanodrop ND-1000 spectrophotometer (Nanodrop Technologies) and its quality was assayed by measuring the A260/A280 ratio, in the range of 1.8-2.0.

one µg of the total RNA was reverse-transcribed with the help of the RevertAid RT Kit (Thermo Fisher Scientific, Waltham, Massachusetts, USA). Quantitative real-time reverse-transcription PCR was carried out on an ABI 7500 RT PCR thermal cycler (ABI 7500, NY, USA) using a SYBR Premix Ex Taq II (Tli RNaseH Plus) kit (TaKaRa Bio Company Kusatsu, Shiga Japan), following the manufacturer’s instructions. Primer sequences were designed for all the genes with the help of the Primer Express, and then the Primer-BLAST (NCBI) was used to check their specificity. Using the 2-∆∆CT method, the relative abundance of mRNA transcripts was calculated utilizing the human Beta 2 microglobulin (hβ2M). Each measurement was performed in triplicate. The primer sequences of the real-time PCR (IL12A NM_000882.3, IL12B NM_002187.2, IL23A NM_016584.2, IL27 NM_145659.3, and B2M NM_004048.2) are listed in Table 1.

**Statistical analysis**

In order to evaluate the expression levels of the IL-12A, IL-12B, IL-23A, and IL-27 among the target subjects, the Relative Quantitative (RQ) qPCR technic was used. In this method the expression value of a gene is shown as the fold change. The values that are less than 0.5 mean decrement in comparison with the calibrator and 0.5 < RQ < 1.9, depicts no change. The values which are higher than 2 illustrate the increment in the expression of the target genes as compared to control. All the data are represented as the mean ± S.D (standard deviation) and executed using the GraphPad Prism software (version 6, USA). Statistical significance was achieved when P < 0.05. 

**Table 1:** Primer sequences used for Real-time PCR

Primer	Sequence (5’–>3’)
IL-12A	F CCA GAA GGC CAG ACA AAC TC R GCC AGG CAA CTC CCA TTA G
IL-12B	F GCG GAG CTG CTA CAC TCT C R CCA TGA CCT CAA TGG GCA GAC
IL-23A	F CTC AGG GAC AAC AGT CAG TTC R ACA GGG CTA TCA GGG AGC A
IL-27	F AGA CGG CAG GCG ACC TTG R GCG AGA TGC AGG CTG ACT G
hβ2M	F TGC TGT CTC CAT GTT TGA TGT ATC T R CTC TGC TCC CCA CCT CTA AGT

**Table 2:** The mean age and BMI of the subjects in 2 phases of the IBD

Variable	Phase Disease	N	Minimum	Maximum	Mean ±SD
Age (mean ±SD)	Flare up	28	20.00	61.00	35.1429±11.02
	Remission	21	18.00	61.00	37.0476±13.85
BMI	Flare up	28	11.73	38.57	24.3067±5.53
	Remission	21	17.36	38.57	27.4307±6.02

**Table 3:** Description of characterization of the IBD patients

Variable		Frequency	Percent
Flare up	Phase disease	28	57.1
Remission		21	42.9
UC	IBD Type	43	87.8
CD		6	12.2
Yes	Family history	2	4.1
No		47	95.9
Female	Gender	29	59.2
Male		20	40.8
Never Used	Alcohol	40	81.6
Current Used		5	10.2
Used		4	8.2
Never Used	Cigarette	46	93.9
Current Used		1	
Used			

## Results

In this study, 49 IBD patients, 29 female and 20 male were evaluated. All cases were Iranian patients and patients from other nations were excluded. The clinical characteristics of the study subjects are shown in Table 2. The average age in the patients with remission and flare-up phases was 37.04 and 35.14, respectively. The average BMI in remission cases was 27.43, whereas the BMI in cases with flare-up was 24.30 (Table 3).

The examination of the expression levels in the target genes among the remission and flare-up phases of the IBD patients showed marked increased in the mRNA contents of IL-12A (P=0.003) (Figure 1) and IL-12B (P=0/0002) in the remission phase as compared to the flare-up period. However, the expression pattern of IL-23A and IL-27 did not change significantly among the studied cases.

**Figure:**
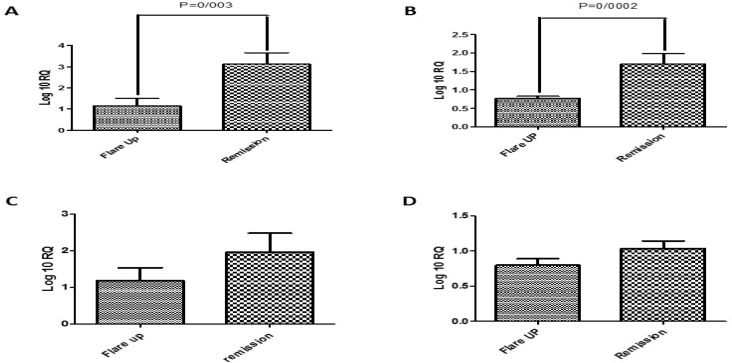


**Figure:**
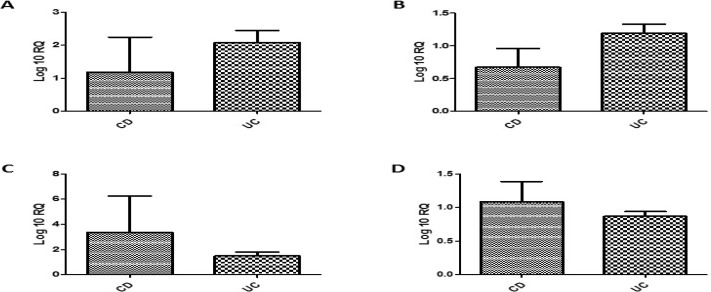


**Figure:**
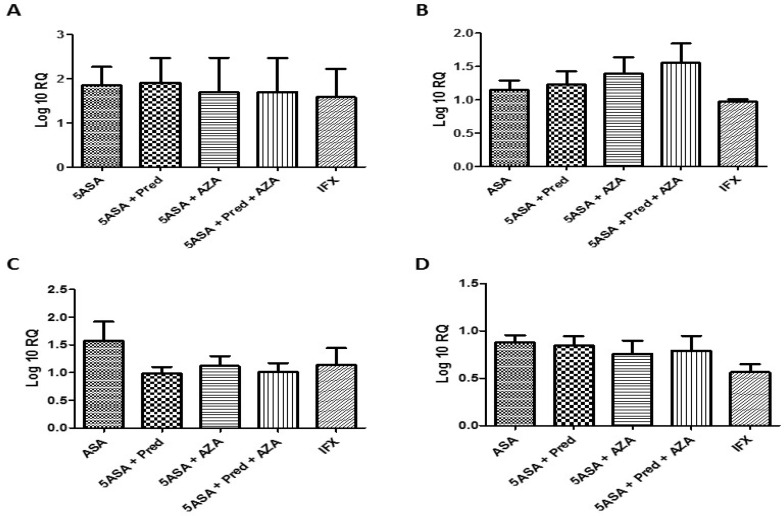


The results of the comparison between the UC patients and the CD ones proved that the differences in the expression levels of the target interleukins genes were not statistically meaningful in either of these groups (Figure 2).

As shown in figure 3, analyzing the mRNA levels of the target genes among the IBD cases based on the type of the drugs intake revealed that any of these treatments was not able to efficiently change the expression behavior of the targeted inflammatory cytokines.

## Discussion

The IBD disease appeared mainly among the UC and CD models. Despite their similarity, each of them has been defined by their own clinical specifications and behavior signatures (1). Considering the unknown etiology of this disease, both of them are affected by the genetic potential of the host, the intensity of the immune responses, and environmental parameters, such as the composition of intestinal microflora (18). 

Currently, UC and CD are the main causes of the IBD disorder in developing countries. Due to the growing prevalence of IBD in the world, it is considered as a high priority issue in health care system (19). The incidence of IBD has been estimated to 1 case per 200 in northern Europe (1). Meanwhile, the overall rate of IBD in Oceania, except Australia and New Zealand, is slowly decreased. Although the epidemiology data in Japan, South Korea, and Hong Kong show marked increase in the frequency of IBD, the actual incidence of this disease in the rest of the Asian countries is still unknown (1, 2). 

Normally, the majority of IBD cases occur between the second and third decade of life. Almost 5 to 30 percent of the UC patients have experienced the disease before 20 years of age. Also, the frequency of the disease was similar among men and women (20). Till this date, the accurate proportion of IBD in the Iranian population is not clarified. However, in 2005, Aghazadeh *et al*. described the demographic features and clinical characteristics of IBD in Iranian patients but the pattern of IBD process in Iranian patient were not evaluated (21, 22). The authors reported that the annual incidence of IBD was expected to increase in the following years and showed the urgent need for setting up an effective diagnosis and prognosis method (21).

Schmidt *et al*. described that p19 part of IL-23 was significantly increased in inflamed mucosa of colon in CD patients and also a lesser extent in UC patients but not in SC patients. Increased level of transcript subunit p19 of IL-23 in CD correlated with the severity of lesions in mucosa. All of both subunits of IL-27, p28 and EBI3 transcripts were significantly elevated only in active CD (23). Imielinski *et al.* in a study on 30 individuals with early-onset Crohn's disease found that the Colonic tissue gene expression of IL-27 was significantly lower than in 11 healthy controls (24). Mohammadi *et al.* showed that serum IL-23 levels were increased in patients with ulcerative colitis compared to normal control. They also described that patients in mild group have Lower serum IL-23 level and ESR compared with those in severe group. Also they demonstrated that in patients with UC in compared with control groups, serum IL-23 was increased. Serum IL-23 was associated with disease activity in UC patients (25).

Liu *et al.* in their study confirmed that the levels of IL-23 were higher in IBD specimens than in normal controls. The levels of IgA and IL-10 were negatively correlated with the infiltration of inflammatory cells in the colonic mucosa in IBD patients, The production of IL-10 by mononuclear cells of lamina propria was lower in the IBD group than in the control group, and these levels could be enhanced by blocking IL-23 (26). Kvedaraite *et al.* in a study collected blood and tissue biopsies from untreated pediatric patients undergoing colonoscopy for suspected IBD. qPCR analysis of IL-23p19 mRNA in tissue biopsies showed higher relative expression of IL-23 mRNA in IBD versus non-IBD patients (27). Furuzawa *et al.* studied on Fifty four patients with IBD which included 27 active UC, 12 inactive UC, 10 active CD, and 5 inactive CD the result also showed that IL-27 gene expression was significantly higher in active UC versus inactive UC group. The IL-27 mRNA expression was increased in patients with active CD compared with inactive CD disease.The percentage of IL-27 immunoreactive cells was higher in active UC versus active CD patients and non-inflamed tissue controls. The IL-27 was significantly elevated in active UC and CD patients, and it was associated with severity of disease (28). 

In our study, the samples got from PBMC in both CD and UC patients in two different phases of IBD. Many investigation were done on IL-12 family gene expression in samples of tissue mucosa in IBD compared to control healthy groups. The results were in a variation but the novelty of our study refers to noninvasive pattern for getting the tissue samples for evaluating a prognostic and diagnostic panel in follow up of IBD patients. The expression status of the interleukins genes IL-12A, IL-12B, IL-23A, and IL-27 were examined in a group of Iranian IBD patients, during the remission and active phases. As observed in the obtained results, the expression level of IL-12A and IL-12B increased in a meaningful fashion (P=0.003 and P=0/002, respectively) among the remission compared to Flare up phase. Conversely, there were no significant differences in the mRNA level of IL-23A and IL-27 among the groups that were studied. The expression patterns of the target genes were similar among the UC and CD cases. Also, the type of drug treatment given to the patients had no effect on the expression levels of the target genes (29).

In conclusion, this study proves that the expression levels of IL12 family as the important interleukins genes are different in two phases of IBD: the remission and flare-up. They also stimulate multiple immune system responses in patients. In the other hand, the development of various new therapies that have different mechanisms of action there is an interest in better characterizing patients and selecting those who may respond preferentially to specific treatments. So, the examination of specific biomarkers and cytokines such as IL-12A and IL-12B levels may be considered to design better diagnostic and prognostic panel to personalize the care of IBD patients. Future studies need to clarify the role of biomarkers and cytokines fluctuation patterns in active a non-native phase of disease.
